# Targeting Ferroptosis to Treat Cardiovascular Diseases: A New Continent to Be Explored

**DOI:** 10.3389/fcell.2021.737971

**Published:** 2021-08-30

**Authors:** Fangze Huang, Ronghua Yang, Zezhou Xiao, Yu Xie, Xuefeng Lin, Peng Zhu, Pengyu Zhou, Jun Lu, Shaoyi Zheng

**Affiliations:** ^1^Department of Cardiovascular Surgery, Nanfang Hospital, Southern Medical University, Guangzhou, China; ^2^Department of Burn Surgery, The First People’s Hospital of Foshan, Foshan, China

**Keywords:** ferroptosis, cardiovascular disease, iron metabolism, cardiomyopathy, myocardial infarction, myocardial ischemia/reperfusion injury, heart failure, lipid peroxidation

## Abstract

Cardiovascular diseases, including cardiomyopathy, myocardial infarction, myocardial ischemia/reperfusion injury, heart failure, vascular injury, stroke, and arrhythmia, are correlated with cardiac and vascular cell death. Ferroptosis is a novel form of non-apoptotic regulated cell death which is characterized by an iron-driven accumulation of lethal lipid hydroperoxides. The initiation and execution of ferroptosis are under the control of several mechanisms, including iron metabolism, glutamine metabolism, and lipid peroxidation. Recently, emerging evidence has demonstrated that ferroptosis can play an essential role in the development of various cardiovascular diseases. Recent researches have shown the ferroptosis inhibitors, iron chelators, genetic manipulations, and antioxidants can alleviate myocardial injury by blocking ferroptosis pathway. In this review, we systematically described the mechanisms of ferroptosis and discussed the role of ferroptosis as a novel therapeutic strategy in the treatment of cardiovascular diseases.

## Introduction

Cardiovascular diseases (CVDs) are the main cause of death and disability in developing and developed countries, including coronary artery disease, heart failure, aortic aneurysm and dissection, peripheral arterial disease, stroke, arrhythmia, and heart valve disorders (Graham et al., 2007; Arnett et al., 2019). In 2019, the prevalence of CVDs was up to 523 million, and the number of CVDs-related deaths reached 18.6 million (Roth et al., 2020). The pathogenesis of CVDs has been found correlated with the death of cardiac and vascular cells (Whelan et al., 2010; Moe and Marín-García, 2016; Del Re et al., 2019).

Fundamentally, cell death is divided into two different types: accidental cell death (ACD) and regulated cell death (RCD) (Tang et al., 2019). ACD can be triggered by unexpected attacks and injury resulting from any possible control molecular mechanisms (Galluzzi et al., 2015). On the contrary, RCD, also known as programmed cell death (PCD) in physiological conditions, involves precise signaling cascades that are executed by genetically defined effector molecules with unique immunological, functional, and biochemical consequences (Galluzzi et al., 2015). As a kind of adaptive response to restore cellular homeostasis, RCD can be modulated by inhibiting the transduction of lethal signals and improving the capacity of cells to adapt to stress (Taylor et al., 2008; Fuchs and Steller, 2011; Rubinsztein et al., 2012). Apoptosis is a classic form of RCD, which can also be defined as programmed necrosis that functions as a homeostatic mechanism to maintain cell populations in tissues and a defense mechanism in immune reactions (Norbury and Hickson, 2001). Nowadays, more and more non-apoptotic forms of RCD have been shown to exert a significant influence on the occurrence and progress of diseases, such as necroptosis, pyroptosis, and autophagy-dependent cell death (Fearnhead et al., 2017; Tang et al., 2019).

Ferroptosis is an iron-dependent form of non-apoptotic RCD proposed by Dixon et al. (2012). It has been demonstrated that ferroptosis is essentially a process of overwhelming, iron-dependent accumulation of lethal lipid reactive oxygen species (ROS) (Dixon et al., 2012). Ferroptosis is morphologically and mechanistically distinguishable from other forms of RCD (Table 1).

**TABLE 1:** Characteristics of different types of regulated cell death.

Type	Morphological features	Biochemistry	Activation approach	Regulated genes
**Ferroptosis**	Smaller mitochondria with condensed mitochondrial membrane densities; reduction or vanishing of mitochondria crista, outer mitochondrial membrane rupture; normal nucleus	Iron-dependent lipid peroxidation, accumulation of iron and ROS	Iron overload, decreased cystine uptake, GSH depletion	Positive: VDAC2/3, RAS, NOX, TfR1, p53, CARS, ACSL4, NCOA4, GLS2s Negative: GPX4, NRF2, HSPB1/5, SLC7A11
**Apoptosis**	Plasma membrane blebbing without rupture; retraction of pseudopods; chromatin condensation and nuclear fragmentation; formation of apoptotic bodies	Caspase activation; Oligonucleosomal DNA fragment; phosphatidylserine exposure	Activated death receptor	Positive: initiator caspase (CASP2/8/9/10), effector caspase (CASP3/6/7), BCL2 family (BAX, BOK, BAK1, BBC3, BID, PMAIP1, and BCL2L11) and TP53, p53 Negative: Bcl-2 family
**Necroptosis**	Rupture of plasma membrane; cell swelling; moderate chromatin condensation; release of cell contents	Drop in ATP levels; RIPK1, RIPK3 and MLKL phosphorylation; ROS production; DAMPs release	TNF-α plus pan-Caspase inhibitor co-treatment; HSV-1 infection	Positive: RIPK1/3, MLKL
**Autophagy**	Accumulation of double-membraned autophagic vesicle	LC3-I to LC3-II conversion, p62 degradation	Nutritional deficiencies, oxidative stress	Positive: ATG5/7, Beclin 1, AMPK Negative: mTOR
**Pyroptosis**	Plasma membrane rupture, release of cell contents, unaffected mitochondrial integrity	Activation of caspase-1 and GSDMD, GSDMDN–induced pore formation, IL-1β release	Activation of inflammasomes	Positive: CASP1/11, GSDMD Negative: PKA, ESCRTIII, GPX4

Recently, several studies have indicated that ferroptosis contributes to the stress-induced deaths of cardiac and vascular cells (Gao et al., 2015; Fang et al., 2019; Wang and Tang, 2019). It has been pointed out that targeting ferroptosis can serve as a feasible approach for preventing cardiomyocyte death and managing cardiac pathologies (Ravingerová et al., 2020; Wu et al., 2021). In this review, we will describe the mechanism of ferroptosis and discuss the role of ferroptosis in the treatment of CVDs, thereby providing a novel therapeutic strategy for CVDs in the future.

## Overview of Ferroptosis

Dolma et al. (2003) found that erastin, a compound lethal to cells expressing RAS^*v12*^, could selectively initiate a cell death procedure, which displayed no apoptotic features such as fragmented nuclei, DNA laddering, and activated caspase 3. Moreover, it was demonstrated by Yang and Stockwell (2008) that an inhibitor of glutathione peroxidase 4 (GPX4) named RAS-selective lethal 3 (RSL3), could induce rapid and non-apoptotic cell death in oncogenic RAS containing tumorigenic cells. This kind of non-apoptotic cell death could be prevented by genetic inhibition of cellular iron uptake or pharmacological iron chelation, which could not be completely reversed by the suppression of necrosis, apoptosis, autophagy, and necroptosis (Yagoda et al., 2007; Yang and Stockwell, 2008). Dixon et al. (2012) discovered that erastin triggered an iron-dependent accumulation of ROS and led to this novel non-apoptotic cell death, defined as *ferroptosis*. It was indicated that ferroptosis was induced by erastin through the inhibition of cystine uptake by the cystine/glutamate antiporter, which suppressed antioxidant defenses due to glutathione reduction (Dixon et al., 2012). This identified ferroptosis as a novel form of RCD and distinguished it from the other types of non-apoptotic cell death.

The occurrence of ferroptosis relies on enormous cellular iron and lipid hydroperoxide, which subsequently induce overwhelming lipid accumulation in cells and interfere with the homeostasis of redox reactions, thus promoting cell death (Xie et al., 2016; Dixon, 2017; Stockwell et al., 2017; Zhai et al., 2021). Ferroptosis can initiate the Fenton reaction and other peroxidation with excessive iron, which can convert the product of mitochondrial oxidative respiration, hydrogen peroxide, into hydroxyl-free radical under the catalysis of ferrous ion. This procedure leads to the accumulation of ROS that can destroy the integrity of cell membrane (Dixon et al., 2012; Qiu et al., 2020). The representative morphology of ferroptosis is shrunken mitochondria, which primarily exhibits increased membrane density, cristae degeneration, and breakdown (Xie et al., 2016). But few other significant morphological changes can be observed before the procedure of ferroptosis (Xie et al., 2016).

## Mechanism of Ferroptosis

Ferroptosis is a complex process regulated by various mechanisms. Peroxidation of phospholipids (PLs) with polyunsaturated fatty acyl tails is considered as the primary driving factor for ferroptosis (Dixon et al., 2012). It has been demonstrated that the occurrence of ferroptosis requires three critical events: iron accumulation, glutathione (GSH) depletion, and lipid membrane oxidation (Bertrand, 2017). Based on those events and other related mechanisms, a large number of reagents have been discovered to induce or inhibit ferroptosis (Table 2). These mechanisms and regulators will be discussed below.

**TABLE 2:** Common inducers and inhibitors of ferroptosis and their functioning mechanisms.

	Reagents	Mechanisms	References
Inducer	Erastin, Sulfasalazine	Inhibit system x_*c*_^–^ and cause GSH depletion	Dixon et al., 2012
	Sorafenib		Dixon et al., 2014
	Glutamate		Zhang et al., 2019
	INF-γ	Downregulate expression of system x_*c*_^–^	Zitvogel and Kroemer, 2019
	RSL3, ML162	Inhibit GPX4 and lipid peroxidation	Yang et al., 2014
	FIN56	Deplete CoQ^10^ and degrade GPX4	Gaschler et al., 2018
	FINO_2_	Deactivate GPX4 and oxidate iron	
	Statins	Inhibit mevalonate pathway to prevent CoQ^10^ synthesis	Viswanathan et al., 2017
Inhibitor	Ferrostatin-1	Block lipid peroxidation	Dixon et al., 2012
	Liproxstatins		Friedmann Angeli et al., 2014
	Glutathione		Schreiber et al., 2019
	CoQ^10^		
	Vitamin E, α-Tocopherol	Suppress LOXs	Kagan et al., 2017
	Iron chelator	Deplete iron	Li N. et al., 2020
	Troglitazone, Rosiglitazone, Pioglitazone	Inhibit ACSL4	Doll et al., 2016

*GSH, glutathione; GPX4, glutathione peroxidase 4; RSL3, RAS-selective lethal 3; LOX, lipoxygenase; ACSL4, acyl-CoA synthetase long-chain family member 4.*

### Iron Metabolism and Homeostasis

It has been proposed that the free radical generation by iron is a pivotal event during ferroptosis. A study in 2015 revealed that under amino acid starvation, ferroptosis occurred by incubating mouse embryonic fibroblasts in serum containing transferrin (Tf) (Gao et al., 2015). Moreover, the rate of cell death was reduced when Tf receptor expression was inhibited with RNA interference or when embryonic fibroblasts were incubated in the presence of iron-free Tf (Gao et al., 2015). These results demonstrate that iron metabolism is relevant to ferroptosis.

Concentrations and homeostasis of iron *in vivo* are regulated by various mechanisms, which control the metabolism, transfer, uptake, and export, as well as intracellular storage of iron (Ward and Kaplan, 2012; Drakesmith et al., 2015). Circulating iron exists in the form of ferric iron (Fe^3+^) by the combination with Tf (Wang and Pantopoulos, 2011; Xie et al., 2016). The import of Fe^3+^ into cells is implemented through the membrane protein, Tf receptor 1 (TfR1) (Wang and Pantopoulos, 2011; Xie et al., 2016). After the import, Fe^3+^ is located in the endosome and then reduced to ferrous iron (Fe^2+^) by the ferrireductase activity of STEAP3 (Hentze and Kühn, 1996; Wang and Pantopoulos, 2011; Xie et al., 2016). Subsequently, Fe^2+^ releases from the endosome into a labile iron pool (LIP) in the cytoplasm, mediated by divalent metal transporter 1 (DMT1) (Wang and Pantopoulos, 2011; Xie et al., 2016). The rest, excess iron is stored in ferritin (FT), an iron storage protein complex consisting of FTL and FTH1 subunits (Wang and Pantopoulos, 2011). The export of iron from cells is mediated by the membrane protein ferroportin (FPN), which can oxidize Fe^2+^ to Fe^3+^ (Xie et al., 2016). FPN is regulated by hepcidin, a 25-amino acid protein released mainly in the hepatocytes, which promotes the internalization and degradation of FPN when iron concentration is high (Ravingerová et al., 2020).

**FIGURE 1:**
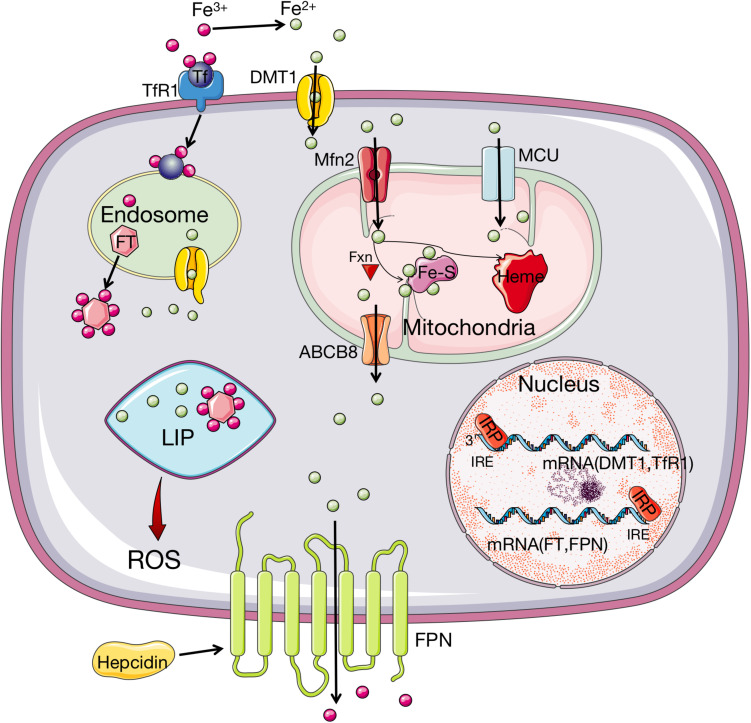
Iron metabolism and homeostasis in organism. Fe^3+^ is imported into cells through Tf recognized by TfR1 while the uptake of Fe^2+^ is implemented through DMT1. Then Fe^3+^ is converted to Fe^2+^ in endosome, which is released from endosome by DMT1. In cell, iron is stored by binding FT, with a small amount staying at LIP. When the amount of iron in LIP increases to the homeostatic limits, ROS will be produced. The transport of iron to mitochondria is via MFRN and MCU. In mitochondria, iron can be synthesized to heme, or Fe–S under the participation of Fxn. The export of iron from mitochondria is through ABCB8 transporter and from cells is mediated through FPN. FPN can oxidize Fe^2+^ to Fe^3+^, which is regulated by hepcidin. Iron homeostasis is controlled by IRP1/2, which can bind to IRE sites of mRNA of DMT-1, TfR1, FT, and FPN to regulate the influx and efflux of iron depending on the iron availability. When the intracellular iron level is low, IRPs bind to the 3′ UTR site of the mRNA of DMT1 and TfR1 to stabilize their transcript, whereas the 5′ UTR site mRNA of FT and FPN is also bound by IRPs to inhibit translation. Fe^3+^, ferric; Tf, transferrin; TfR1, transferrin receptor 1; Fe^2+^, ferrous; DMT1, divalent metal transporter 1; FT, ferritin; LIP, labile iron pool; ROS, reactive oxygen species; MRFN, mitoferrin; MCU, mitochondrial calcium uniporter; Fe–S: iron–sulfur cluster; Fxn, frataxin; FPN, ferroportin; ABCB8, adenosine triphosphate (ATP)-binding cassette subfamily B member 8; IRP1/2, iron regulatory protein 1/2; IRE, iron responsive elements.

Iron homeostasis is regulated by a post-transcriptional mechanism by the interaction of iron regulatory proteins (IRP) 1 and 2 with iron-responsive elements (IRE) on mRNA of respective genes, which modulate the synthesis of essential iron metabolism proteins that participate in iron uptake, storage, and release (Hentze and Kühn, 1996). Under conditions of low cellular iron concentration, IRP stabilizes the mRNA of TfR1 and DMT-1 to promote iron influx (Haddad et al., 2017; Paterek et al., 2019). Meanwhile, IRP prevents mRNA of FPN 1 and FT from translating to inhibit iron efflux and storage (Haddad et al., 2017; Paterek et al., 2019). This procedure results in a stable LIP, which is a crossroad of cellular iron metabolism. As a pool of chelatable and redox-active iron complexes, LIP is an intermediate or transitory pool between extracellular iron and cellular iron associated with proteins (Kakhlon and Cabantchik, 2002). When the concentration of the LIP increases to the homeostatic limits, severe oxidative damage occurs by initiating the Fenton reaction and other peroxidation, which will produce ROS and induce lipid peroxidation (Kakhlon and Cabantchik, 2002; Doll and Conrad, 2017; Qiu et al., 2020). The procedure of iron metabolism is presented in Figure 1.

Besides the iron homeostasis, it has been proposed that ferritinophagy, a process in which FT is selectively sequestered into autophagosomes and delivered to lysosomes for degradation, can trigger ferroptosis by promoting the accumulation of iron and ROS (Gao et al., 2015; Hou et al., 2016; Tang et al., 2018). Ferritinophagy can control iron availability and influence other proteins involving in ferroptosis to enhance the sensitivity of ferroptosis (Mancias et al., 2014; Sun et al., 2015; Hou et al., 2016; Tang et al., 2018). Therefore, iron metabolism and ferritinophagy can serve as the potential regulated targets for ferroptosis.

### Abnormal Glutaminolysis

Amino acid metabolism is related to the regulation of ferroptosis (Angeli et al., 2017). Glutamine naturally exists at high concentrations in human tissues and plasma, and its degradation through glutaminolysis provides materials for the tricarboxylic acid cycle and essential biosynthetic processes such as lipid biosynthesis. This indicates that glutaminolysis is capable of reducing the accumulation of ROS and thus the occurrence of ferroptosis. It has been reported that ferroptosis can be initiated both by direct inhibition of GPX4, and an essential intracellular antioxidant, GSH, which are the crucial proteins in glutaminolysis (Wu et al., 2021).

**FIGURE 2:**
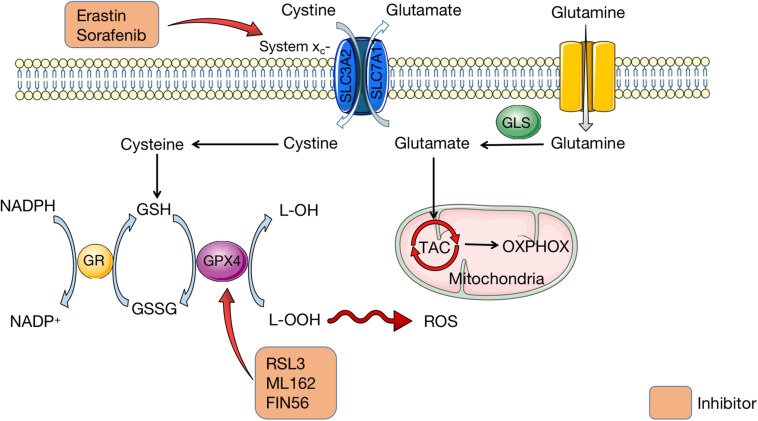
Amio acid metabolism in ferroptosis. Containing two subunits (SLC3A2 and SLC7A11), system x_*c*_^–^ is the cystine/glutamate reverse transporter on cell membrane, which transports cystine into cells and glutamate outside cells. Glutamate is transferred from glutamine, which is mediated by GLS. Then glutamate is imported into mitochondria for TAC to synthesize OXPHOX. In cells, cystine is degraded to cysteine for GSH synthesis. GSH is conversed to GSSG under the catalysis of GPX4, synchronized with conversion of L-OOH to L-OH. Meanwhile, NADPH is transferred to NADP+, which is catalyzed by GR. Erastin and sorafenib are inhibitors of system x_*c*_^–^ to affect the amio acid metabolism. RSL3, ML162, and FIN56 can inhibit GPX4 and thus lead to excessive L-OOH in cells, which causes ROS accumulation. GLS, glutaminase; TAC, tricarboxylic acid cycle; OXPHOX, oxidative phosphorylation; GSH, glutathione; GSSG, glutathione disulfide; GPX4, glutathione peroxidase 4; NADPH, nicotinamide adenine dinucleotide phosphate; GR, glutathione reductase; RSL3, RAS-selective lethal 3; ROS, reactive oxygen species.

When ferroptosis was first defined, it was induced by erastin which inhibited cystine uptake by the cystine/glutamate antiporter, leading to suppressed antioxidant defenses due to GSH reduction (Dixon et al., 2012). This is the classic pathway to initiate ferroptosis. It is indicated that erastin inhibits cystine uptake which is mediated by system x_*c*_^–^, a member of the heterodimeric amino acid transporter family (Dixon et al., 2012). Containing two subunits (SLC3A2 and SLC7A11), system x_*c*_^–^ is the cystine/glutamate reverse transporter, which is expressed on cell membrane and capable of maintaining redox homeostasis (Bentea et al., 2020; Kim et al., 2020; Li et al., 2021). System x_*c*_^–^ can transport glutamate into the extracellular space and meanwhile cystine into the cell on an equal ratio. After imported by system x_*c*_^–^, cystine is reduced and degraded to cysteine, which is utilized to synthesize antioxidant GSH (Bentea et al., 2020; Kim et al., 2020). Under the catalysis of GPX4, GSH converses to glutathione disulfide (GSSG) (Mirochnitchenko et al., 2000; Yang et al., 2014). In the meantime, free hydrogen peroxide is converted to water, or lipid hydroperoxides (L–OOH) are reduced to lipid hydroxy derivative (L–OH) by GPX4 (Mirochnitchenko et al., 2000; Yang et al., 2014). These procedures are essential for the maintenance of cellular redox homeostasis. When glutamine is absent or glutaminolysis is inhibited, cystine starvation and blockage of cystine import fail to induce the accumulation of ROS, lipid peroxidation, and ferroptosis (Gao et al., 2015; Stockwell et al., 2017). On the contrary, GSH depletion leads to the inactivation of GPX4 and thus produces excessive ROS (Yang et al., 2014). Hence, it is reasonable to hypothesize that system x_*c*_^–^ and GPX4 serve as negative regulators of ferroptosis. Figure 2 shows how amino acid metabolism is related to ROS production in organism.

Nevertheless, not all routes of glutaminolysis promote ferroptosis. The first step of glutaminolysis involves the conversion of glutamine into glutamate which is catalyzed by the glutaminases GLS1 and GLS2. Only GLS2 takes part in the initiation of ferroptosis, though GLS1 and GLS2 are structurally and enzymatically similar (Gao et al., 2015). The GLS2 gene is a transcriptional target of the tumor suppressor p53, and upregulation of GLS2 can promote p53-dependent ferroptosis (Jennis et al., 2016). Therefore, a precise target for inhibiting ferroptosis in amino acid metabolism still requires further researches.

### Lipid Peroxidation

Lipid metabolism is intimately associated with cell death because it compromises membrane structural integrity, exerts downstream cytotoxic effects, and is involved in suicide signaling cascades (Reed, 2011; Yin et al., 2011; Ayala et al., 2014). As mentioned above, the inhibition of GPX4 causes lethal accumulation of lipid peroxides and thus leads to ferroptosis. This pathway has been demonstrated to mostly affect polyunsaturated fatty acid (PUFA), which contains bis-allylic hydrogen atoms that can be readily abstracted (Yang et al., 2016). Therefore, the abundance and localization of PUFA determine the degree of lipid peroxidation in cells, and the level of ferroptosis. PUFA must be esterified into membrane PLs and oxidated to become ferroptotic signals (Kagan et al., 2017). Also, accumulation of PL hydroperoxides has been detected in ferroptosis, including phosphatidylcholine, cardiolipin, and phosphatidylethanolamine (Yang et al., 2016). Hence, it has been proposed that PLs containing PUFA are the major substrates of ferroptotic lipid peroxidation and membrane damage is an essential event in ferroptosis (Friedmann Angeli et al., 2014; Yang et al., 2016; Kagan et al., 2017).

Recently, several lipidomic studies proposed that phosphatidylethanolamines (PEs) containing arachidonic acid (AA) or its elongation product, adrenic acid are pivotal PLs that undergo oxidation and actuate ferroptosis (Doll and Conrad, 2017; Kagan et al., 2017). As a result, coenzyme-A-derivatives that participate in the synthesis of these PUFAs and their insertion into PLs are required to produce ferroptotic signals. Two lipid metabolism-associated enzymes, lysophosphatidylcholine acyltransferase 3 (LPCAT3) and acyl-CoA synthetase long-chain family member 4 (ACSL4) are proved to participate in the biosynthesis and restructuring of PUFA-PEs in cell membrane (Dixon et al., 2015). In fact, ACSL4 acylates AA and then LPCAT3 catalyzes the acylated AA into membrane PLs, which increases the oxidization of sensitive fatty acids such as PUFA in the membrane and eventually causes lipid peroxidation (Xie et al., 2016).

**FIGURE 3:**
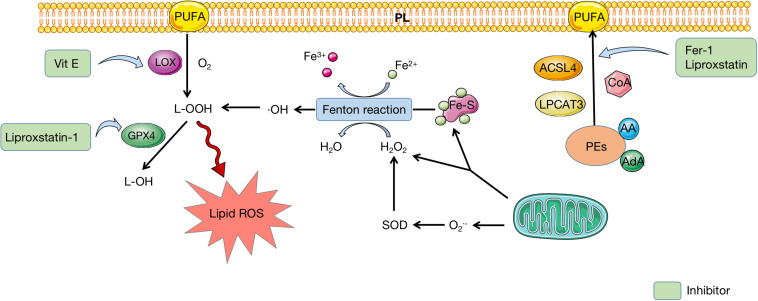
Lipid metabolism in ferroptosis. PEs containing AA or AdA are fatty acid substrates, which are synthesized to PUFA under the catalysis of ACSL4, LPCAT3, and CoA. PUFA can insert to PL and be oxidated to produce L-OOH, which is catalyzed by LOX. Mitochondria provides Fe-S and H_2_O_2_ to participate in Fenton reaction, during which iron is oxidated and H_2_O_2_ is deoxidated. Reactive free radicals, ⋅OH, is produced and eventually conversed into L-OOH, which leads to accumulation of lipid ROS. GPX4 can mediate the transformation of L-OOH to L-OH, which can be inhibited by liproxstatin-1. Fer-1 and liproxstatin are inhibitors of PUFA synthesis, while Vit E can inhibit LOX to prevent PUFA oxidation. PEs, phosphatidylethanolamines; AA, arachidonic acid; AdA, adrenic acid; PUFA polyunsaturated fatty acid; PL, polyunsaturated fatty acid; LOX, lipoxygenase; Fe-S, iron–sulfur cluster; SOD, superoxide dismutase; ROS, reactive oxygen species; GPX4, glutathione peroxidase 4; Fer-1, ferrostatin-1; Vit E, vitamin E.

The formation of lipid hydroperoxides is demonstrated to be associated with enzymatic reactions and autoxidation catalyzed by lipoxygenase (LOX) (Yang et al., 2016). In ferroptosis, LOXs can promote the di-oxygenation of free and esterified PUFA to catalyze lipid peroxidation directly (Kuhn et al., 2015; Yang et al., 2016). The first step in LOXs catalysis is the abstraction of a labile hydrogen atom from a bis-allylic position on PUFA. Next, molecular oxygen adds to the intermediate carbon-centered radical to produce a peroxyl radical, which is then deoxidated by the enzyme to yield the hydroperoxide product (Kuhn et al., 2015). These products subsequently react with other PUFAs to pass on a chain reaction of lipid peroxidation, which yields PUFA peroxides and reactive aldehydes that cause cell damage (Yang et al., 2016; Figure 3).

Likewise, the progression of oxygen-driven free radical chain reaction, also named non-enzymatic lipid peroxidation, is believed to be involved in the occurrence of ferroptosis. Non-enzymatic lipid peroxidation includes the generation of early lipid radical L⋅ based on the production of sufficiently reactive free radicals, the oxidation of L⋅ in a chain reaction, and the termination of oxidation by antioxidants (Frank, 1950). Furthermore, lipid peroxidation spontaneously produces PLOO⋅ and PLO⋅, which constantly recruit lipid molecules to free radical reactions and form a lipid peroxidation circle (Davies and Guo, 2014). It is believed that Fenton reaction can also provide the free radicals for lipid peroxidation metabolism, which can be a connecting point between iron metabolism and lipid peroxidation in ferroptosis (Zhai et al., 2021).

## Ferroptosis and CVDs

### Iron Homeostasis in the Heart

**FIGURE 4:**
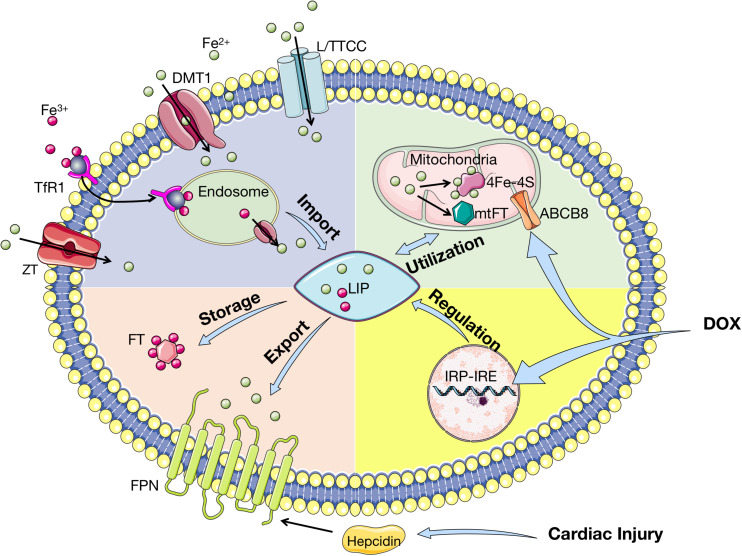
Iron homeostasis in cardiomyocytes. Tf-bound iron is imported into cardiomyocytes via TfR1, whereas Non-Tf-bound iron enters via DMT1, L/TTCC, and ZT. After imported into cardiomyocytes, iron is located in endosome and deoxidated before stored in LIP. In cytoplasm, iron is stored by binding to FT and exported by FPN, which is regulated by hepcidin. Iron is transported into mitochondria for synthesis into 4Fe–4S and mtFT, which stores excessive iron during cardiac injury. ABCB8 is the iron exporter of mitochondria. Cardiac iron homeostasis is regulated by IRP-IRE with upregulation of TfR1 and downregulation of FT and FPN. Cardiac injury affects iron homeostasis by upregulating hepcidin, while DOX influences IRP-IRE and ABCB8. Tf, transferrin; TfR1: transferrin receptor 1; DMT1, divalent metal transporter 1; L/TTCC, L/T-type calcium channel; ZT, Zinc transporter; LIP, labile iron pool; FT, ferritin; FPN, ferroportin; Fe–S, iron–sulfur cluster; mtFT, mitochondrial ferritin; ABCB8, adenosine triphosphate (ATP)-binding cassette subfamily B member 8; IRP, iron regulatory protein; IRE, iron responsive elements; DOX, doxorubicin.

The regulation of iron homeostasis in cardiac myocytes is similar to that of systemic iron homeostasis mentioned above. The import of iron is mediated by TfR1 and the export of iron from cells is implemented via FPN. Different from systemic cells, FPN in cardiomyocytes is regulated by both hepcidins produced by liver and locally in heart. Cardiac hepcidin has important autocrine effects and participates in autonomous regulation of iron in cardiomyocytes. Opposite to systemic hepcidin, loss of cardiac hepcidin upregulates FPN in cardiomyocytes to maintain cellular iron homeostasis (Lakhal-Littleton et al., 2016). It is indicated that iron level in cardiomyocytes is a balance between cellular iron efflux regulated by the cardiac hepcidin/FPN axis and systemic iron availability regulated by the systemic hepcidin/FPN axis (Lakhal-Littleton et al., 2016). In fact, cardiomyocytes are extra sensitive to iron overload with sufficient iron-importing mechanisms and only one export regulator. Nevertheless, either overload or deficiency of iron is harmful to homeostasis of cardiomyocytes. The import, utilization, storage, export, and regulation of iron in cardiomyocytes are presented in Figure 4. Interestingly, mitochondrial ferritin (mtFT) is an *H*-ferritin-like protein to store iron in mitochondria, which is mostly expressed in high oxygen-consuming and high metabolic cells. A recent study has demonstrated that mtFT can protect cardiomyocytes against the oxidative stress caused by cardiac injury via increasing the sensitivity of mitochondria (Li X. et al., 2017). The role of mtFT in iron homeostasis in cardiomyocytes still needs more researches to confirm.

Cardiomyocytes apoptosis can be induced by iron overload through mitochondrial dysfunction, in which increased mitochondrial oxidative stress triggers the release of cytochrome *c* and activates the caspase-dependent apoptotic pathway (Khamseekaew et al., 2017; Kumfu et al., 2018). This can result in iron-overload cardiomyopathy (IOC), which occurs in hemochromatosis and thalassemia major patients (Gulati et al., 2014). Moreover, the European Society of Cardiology currently recommended that the assessment of iron deficiency served as a comorbidity in chronic heart failure (CHF), due to the high prevalence of iron deficiency in patients with CHF (Klip et al., 2013).

### The Role of Ferroptosis in CVDs

#### Ferroptosis and Cardiomyopathy

Several studies have indicated that ferroptosis is involved in many cardiomyopathies, including IOC, diabetic cardiomyopathy (DCM), doxorubicin (DOX)-induced cardiotoxicity, and so on.

As mentioned above, iron overload can destroy cardiac iron homeostasis, thereby leading to IOC through several mechanisms. IOC is regarded as a progressive electromechanical deterioration of the heart, which is the major reason for fatality in hemochromatosis patients (Nakamura et al., 2019). Also, several studies have demonstrated that high concentrations of intracellular iron induce ferroptosis in cardiomyocytes. Baba et al. (2018) verified that excessive iron induced cardiomyocytes ferroptosis as efficiently as erastin and RSL3 by directly incubating isolated mouse cardiomyocytes in ferric citrate. Furthermore, they found that cardiomyocytes treated with both Fe(III)-citrate and ferrostatin-1 (Fer-1), a specific ferroptosis inhibitor, were prevented from Fe(III)-citrate-induced cell death (Baba et al., 2018). This indicated that ferroptosis might play a significant role in the pathophysiological process of IOC, but the mechanism of how ferroptosis was associated with IOC remained unclear and needed further studies.

Diabetic cardiomyopathy, which is characterized by hypertrophy and fibrosis in the heart with the absence of clinical hypertension and coronary artery disease, is one of the most common complications of diabetes (Bugger and Abel, 2014; Parim et al., 2019). It can result in left ventricular remodeling event and subsequently develop into heart failure, which involves various mechanisms such as hyperglycemia, insulin resistance, increased fatty acid oxidation, oxidative stress, myocardial fibrosis and hypertrophy, endothelial dysfunction, myocyte cell death, autonomic neuropathy, arterial stiffness, autophagy, endoplasmic reticulum stress and so on (Coats and Anker, 2000; Fiordaliso et al., 2001; Vinereanu et al., 2003; Brunner et al., 2006; Turan, 2010; Lakshmanan et al., 2013; Riehle et al., 2013; Brahmanaidu et al., 2017; Parim et al., 2019). Nowadays, it has been widely accepted that oxidative stress is the common pathogenesis of diabetic cardiomyopathy, which results from an imbalance between the production of ROS and the antioxidant capacity (Khullar et al., 2010; Huynh et al., 2014). Based on that, a recent research found that knockdown of nuclear factor-erythroid factor 2–related factor 2 (Nrf2), a main regulator of antioxidant defense, selectively suppressed glucolipotoxicity, while Fer-1 and iron chelator deferoxamine inhibited glucolipotoxicity in rat H9C2 cells cultured in high glucose (Zang et al., 2020). Moreover, the expression of ferroptosis markers, cyclooxygenase 2 (Cox2) and glutathione-specific γ-glutamylcyclotransferase 1 (Chac1), was upregulated, while the expressions of GPX4 and ferroptosis suppressor protein 1 (Fsp1) were downregulated in mice with streptozotocin-induced type 1 diabetes (T1D) (Zang et al., 2020). These results indicated that autophagy deficiency caused by diabetes initiated an Nrf2-operated ferroptosis in cardiomyocytes, thereby worsening the progression of diabetic cardiomyopathy. Hence, Nrf2 pathway-mediated ferroptosis should be focused on in the future, since it can be a novel target for treatment of DCM.

DOX is a class of anthracyclines that is commonly used to treat breast cancer, leukemia, and several types of malignancies (Young et al., 1981). Nevertheless, the clinical use of DOX is limited due to its cardiotoxicity, which can induce irreversible degenerative cardiomyopathy and congestive heart failure (Singal and Iliskovic, 1998). Fang et al. (2019) indicated that ferroptosis drove DOX-induced cardiomyopathy, owing to the results that DOX-treated cardiomyocytes in mice showed features of typical ferroptotic cell death and Fer-1 significantly decreased DOX-induced myocardial injury. Besides, by measuring mitochondrial lipid peroxidation to examine the effects of combining Fer-1 with zVAD-FMK (zVAD), an apoptosis inhibitor, in DOX-treated cardiomyocytes, Tadokoro et al. (2020) found that ferroptosis was the main form of RCD and triggered in mitochondria under DOX treatment. Luo et al. (2021) proved that DOX promoted ferroptosis in cardiomyocytes, whereas the administration of Astragaloside IV, an ingredient isolated from astragalus membranaceus, inhibited ferroptosis by activating Nrf2 signaling pathway and increased GPX4 expression (Luo et al., 2021). These studies demonstrated ferroptosis exerted a significant influence on DOX-induced cardiomyopathy and proved the therapeutic validity of inhibiting ferroptosis.

There is also a correlation between ferroptosis and sepsis-induced cardiomyopathy. Li N. et al. (2020) discovered that Fer-1 inhibited LPS-induced lipid peroxidation and injury of H9C2 myofibroblasts, which illustrated that ferroptosis served as a critical mechanism contributing to sepsis-induced cardiac injury. It is also proposed that targeting ferroptosis in cardiomyocytes may be a promising therapeutic strategy for preventing sepsis in the future.

#### Ferroptosis and Myocardial Infarction

Myocardial Infarction (MI) is a clinical term for heart attack, which is commonly caused by myocardial ischemia due to narrow or blocked coronary arteries. MI manifests as the death of cardiomyocytes and the replacement of damaged heart tissues by fibrotic scar tissue, which is unable to compensate for contraction function and thus causes heart failure (Hashimoto et al., 2018). Currently, a variety of regulated cardiomyocyte deaths have been focused on and proved to play an important role in MI (Lee et al., 2003; Del Re et al., 2019). Bulluck et al. (2016) revealed that most of the ST-segment-elevation MI patients with IMH had residual myocardial iron at follow-up, which indicated that residual myocardial iron might be a potential therapeutic target to prevent adverse left ventricular remodeling in reperfused cardiac tissue of MI. By using quantitative proteomic analyses, Park et al. (2019) found that GPX4 and ROS pathway was downregulated significantly in early and middle stages of MI. This substantiates that ferroptosis contributes to cardiomyocyte death during MI under metabolic stress. Also, a recent research found that BTB and CNC homology 1 (BACH1), a regulator in heme and iron metabolism which could repress the transcription of erastin-induced protective genes, aggravates acute MI by promoting ferroptosis (Nishizawa et al., 2020). Tang et al. (2020) discovered autophagy in cardiomyocytes after MI could promote ferroptosis, which could be inhibited by microRNA-30d via binding to ATG5. Moreover, Song et al. (2021) examined the expression of DMT1 *in vivo* and *in vitro* acute MI models, and found that overexpression of DMT1 promoted cardiomyocyte ferroptosis. They also found that exosome of mesenchymal stem cells derived from human umbilical cord blood (HUCB-MSC) suppressed cardiomyocytes ferroptosis to mediate myocardial repair in acute MI by delivering miR-23a-3p (Song et al., 2021). Therefore, inhibition of ferroptosis might be a new insight for repairing cardiomyocyte injury in MI.

#### Ferroptosis and Myocardial Ischemia/Reperfusion Injury

Myocardial ischemia/reperfusion injury (IRI) is mostly caused by oxidative processes with ROS generation as the central pathogenesis. Although ROS from mitochondrion is considered as the primary cause of IRI in myocardium, several researches have purposed the significance of ferroptosis in induction of cardiomyocyte injury. By examining the level of myocardial iron in mouse models of IRI generated by 30-min left anterior descending coronary artery (LAD) ligation, Baba et al. (2018) detected cardiomyocyte death with excessive iron accumulating around the myocardial scar, which proved to be ferroptosis by iron overload. Fang et al. (2019) treated mice with 30 min of myocardial ischemia followed by 24 h of reperfusion and discovered the occurrence of significantly increased cardiac non-heme iron. Also, they found that pretreatment of Fer-1 or iron chelator reduced ischemia/reperfusion-induced cardiac remodeling and fibrosis, with the decrease of cardiac *mt-Cytb* and *mt-Atp6* mRNA levels (Fang et al., 2019). Recently, Chen et al. (2021) discovered an increase in cellular iron levels but decreases in GPX4 activity as well as FTH1 and GSH levels in mice myocardial IRI model. Meanwhile, they found that the knockdown of embryonic lethal-abnormal vision like protein 1 (ELAVL1) could attenuate ischemia/reperfusion-induced ferroptosis by inhibiting autophagy, which was activated by Forkhead box C1 (FOXC1) in cardiomyocytes treated with hypoxia followed by reoxygenation (Chen et al., 2021). Fan et al. (2021) indicated that baicalin inhibited ACSL4-controlled ferroptosis to ameliorate myocardial IRI *in vitro*. Stamenkovic et al. (2021) put forward that increased oxidized phosphatidylcholines (OxPCs) generated in myocardial IRI provoked cardiomyocyte death through ferroptosis. Furthermore, several medications have been proposed to alleviate IRI by interfering with ferroptosis. Lv et al. (2021) proved that Etomidate suppressed ferroptosis in IRI model via upregulation of Nrf2 and heme oxygenase-1 (HO-1) protein expression. Cyanidin-3-glucoside, a kind of anthocyanin, is verified to attenuate myocardial IRI via ferroptosis inhibition by reducing oxidative stress and Fe^2+^ accumulation *in vivo* and *in vitro* (Shan et al., 2021). Ma et al. (2020) demonstrated that USP22 (ubiquitin-specific protease 22), a member of the deubiquitinase family, could inhibit ferroptosis in myocardial IRI via the SIRT1/p53/SLC7A11 association. On the contrary, USP7 is found to protect cardiomyocytes against ferroptosis caused by IRI via activation of the p53/TfR1 pathway (Tang et al., 2021b). Likewise, ferroptosis is demonstrated to be associated with diabetes myocardial IRI. Li et al. discovered that inhibiting ferroptosis reduced endoplasmic reticulum stress and mitigated myocardial damage (Li W. et al., 2020). These results suggested inhibiting ferroptosis could provide significant protection from myocardial IRI. Interestingly, a recent study found no significant changes in ACSL4, GPX4, iron, and malondialdehyde in cardiac ischemia region, whereas increased ACSL4, iron, and malondialdehyde as well as decreased GPX4 were observed in the reperfusion model (Tang et al., 2021a). Hence, it is believed that ferroptosis takes place in the phase of myocardial reperfusion instead of ischemia, which provides a fresh perspective for intervention of ferroptosis in IRI therapy.

Ischemia/reperfusion injury is one of the toughest challenges in heart transplantation, causing considerable sterile inflammation that leads to a high rate of primary graft dysfunction and even mortality in recipients (Kobashigawa et al., 2014). Li et al. (2019) found that Fer-1 reduced levels of hydroperoxy-arachidonoyl-phosphatidylethanolamine, a mediator of ferroptosis, which also repressed the ferroptosis of fibroblasts instead of endothelial cells in heart grafts submitted to IRI. They indicated that ferroptosis coordinated neutrophil recruitment to injured myocardium by promoting adhesion of neutrophils to coronary vascular endothelial cells through a TLR4/Trif/type I IFN signaling pathway (Li et al., 2019). Therefore, inhibition of ferroptosis in donor hearts before transplantation may reduce IRI and improve prognosis.

#### Ferroptosis and Heart Failure

Heart failure (HF), featured by cardiac hypertrophy and fibrosis, is a clinical syndrome, in which pumping function of heart is damaged and cardiac output fails to meet basic metabolic needs (Li et al., 2018; Miller et al., 2019). As loss of terminally differentiated cardiomyocytes is irreversible in HF, early prevention of cardiomyocyte death can maintain cardiac function and decelerate the progression of heart failure. A recent study observed ferroptotic cell death in the rat HF model induced by descending aortic banding (Liu et al., 2018). What’s more, Puerarin, an antioxidant reagent, could reduce iron content and increase ROS elimination, suggesting that puerarin is a promising therapy for HF by inhibiting cardiomyocyte ferroptosis (Liu et al., 2018). By knockdown of TLR4 and NADPH oxidase 4 (NOX4) in HF rats, Chen et al. (2019) discovered a detainment of ferroptosis which was detected by expression of GPX4 and FTH1. Recently, Fang et al. (2020) found that a high-iron diet caused severe cardiac injury, hypertrophic cardiomyopathy, and eventually HF via inducing cardiomyocyte ferroptosis. Also, they revealed that cardiomyocytes deficient of FTH reduced expression of the SLC7A11 from system x_*c*_^–^, whereas overexpressing SLC7A11 selectively in cardiomyocytes increased GSH levels and prevented cardiac ferroptosis (Fang et al., 2020). Wang et al. (2020) indicated mixed lineage kinase 3 (MLK3), a member of MAP3K family, could regulate the JNK/p53 signaling pathway to initiate ferroptosis and cause myocardial fibrosis in the advanced stage of HF, which could be reversed by miR-351. Circular RNA (circRNA) is a new type of non-coding RNA, which is involved in the pathogenesis of cardiovascular diseases such as HF (Kolakofsky, 1976; Wang et al., 2017). Zheng et al. (2021) constructed a circRNA–miRNA–mRNA regulatory network based on competitive endogenous RNA and verified miR-224-5p, downstream target of circSnx12, could downregulate FTH1 expression in HF model. Altogether, these studies illustrate the significance of ferroptosis in cardiac hypertrophy and HF.

#### Ferroptosis and Vascular Injury

Vascular injury, a complicated type of CVDs including aortic dissection and abdominal aortic aneurysm, is caused by multifactorial damages such as genetic variant, diet, and environment. It is widely acknowledged that smoking is one of the main risk factors leading to aortic dissection and the rupture of abdominal aortic aneurysm (Lederle et al., 2003; Kakafika and Mikhailidis, 2007; Kihara et al., 2017). Sampilvanjil et al. (2020) found that cigarette smoke extract (CSE) initiated ferroptosis in vascular smooth muscle cells (VSMCs) rather than endothelial cells by depleting GSH rapidly and reducing the suppression of GPX4 overexpression, which resulted in medial VSMC loss in *ex vivo* aortas. These findings suggest that ferroptosis is the main cause of CSE-induced VSMC death and vascular injury. Further researches are expected to prove the role of ferroptosis in vascular diseases caused by other pathogenic factors such as atherosclerosis.

#### Ferroptosis and Stroke

Ischemic stroke refers to the restriction of blood supply to certain parts of the brain due to the occlusion of the internal carotid, middle cerebral, or vertebral/basilar arteries, which results in activation of ischemic cascade and ultimately cell death (Brouns and De Deyn, 2009; Au et al., 2017). Speer et al. (2013) hypothesized that ferroptosis might cause neuronal death induced by cerebral ischemia and that iron chelators prevented ferroptosis by inhibiting 2-oxoglutarate, oxygen-dependent dioxygenases, and the hypoxia-inducible factor (HIF) prolyl hydroxylases. Tuo et al. (2017) found that ferroptosis inhibition attenuated IRI in a middle cerebral artery occlusion model. More excitingly, they also found that tau-knockout mice were protected against hemispheric IRI, suggesting the tau–iron interaction as a pleiotropic modulator of ferroptosis and ischemic stroke outcome (Tuo et al., 2017). Recently, a research indicated that ACSL4 expression was downregulated in early ischemic stroke and its overexpression exacerbated ischemic cerebral injury, which proposed that ACSL4 expression might be a potential therapeutic target in ischemic stroke (Cui et al., 2021).

Intracerebral hemorrhage (ICH) occurs when a weakened vessel ruptures and bleeds, thereby leading to higher morbidity and mortality than ischemic stroke (Donnan et al., 2010). Hemoglobin/heme released from lysed erythrocytes after ICH is considered as a neurotoxin to induce lethal ROS after being metabolized into free iron and ultimately cause neuronal death (Ward et al., 2014; Xiong et al., 2014). A research showed that inhibition of iron-dependent hypoxia-inducible factor prolyl hydroxylase domain enzymes (HIF-PHDs) protected neurons from hemin-induced toxicity (Karuppagounder et al., 2016). Li Q. et al. (2017) found that Fer-1 prevented neuronal death and reduced iron deposition induced by hemoglobin in organotypic hippocampal slice cultures. Moreover, Zille et al. (2017) indicated that ICH *in vivo* and *in vitro* shared features of ferroptotic and necroptotic cell death, but not caspase-dependent apoptosis or autophagy. Collectively, all these studies suggest that ferroptosis contributes to neuronal death after ICH.

#### Ferroptosis and Arrhythmia

Cardiac arrhythmia can occur in terminal sudden unexpected death in epilepsy (SUDEP), due to a high rate of hypoxic stress induced by convulsions with excessive sympathetic overstimulation that triggers a neurocardiogenic injury (Nashef et al., 2012). Recently, arrhythmia in SUDEP is considered to be associated with iron overload in conditions of cardiac hypoxia. Akyuz et al. (2021) indicated ferroptosis might be a potential intrinsic mechanism that led to fatal cardiac arrhythmia, with hemosiderin observed in the cardiomyocytes in the SUDEP model. However, the deep mechanism of how ferroptosis is involved in arrhythmia remains unclear. Further investigations on this issue are expected to carry on in the future.

## The Application of Ferroptosis in Treatment of CVDs

As mentioned above, ferroptosis plays a significant role in the occurrence and development of various CVDs. Therefore, targeting ferroptosis is proposed as a feasible approach for cardiac protection (Table 3).

**TABLE 3:** Application of ferroptosis for treatment of cardiovascular diseases.

Reagents	Mechanisms	Protective effects	References
Knockdown of Nrf2	Reduce heme degradation	Prevent Hmox1-dependent ferroptosis in DIC.	Fang et al., 2019
Upregulation of GPX4	Decrease lipid ROS levels	Protect cardiomyocytes in MI.	Park et al., 2019
Overexpression of SLC7A11	Increase GSH levels	Mediate cardiac iron homeostasis and prevent hypertrophic cardiomyopathy.	Fang et al., 2020
Fer-1 DXZ	Prevent lipid peroxidation	Maintain the function of mitochondrial and prevent DIC.	Fang et al., 2019
Lip-1	Increase GPX4 protein levels and reduce ROS generation	Reduce myocardial infarct size and ischemia/reperfusion injury	Feng et al., 2019
Vitamin E	Suppress LOX	Inhibit PUFA oxidation and prevent ferroptosis.	Kagan et al., 2017

*DIC, doxorubicin-induced cardiomyopathy; GPX4, glutathione peroxidase 4; ROS, reactive oxygen species; MI, myocardial infarction; GSH, glutathione; DXZ, dexrazoxane; Fer-1, ferrostatin-1; Lip-1, liproxstatin-1; LOX, lipoxygenase; PUFA, polyunsaturated fatty acid.*

Genetic manipulations in ferroptosis signaling pathway have been verified to successfully inhibit ferroptosis and decrease myocardial injury. The knockdown of Nrf2 to reduce heme degradation (Fang et al., 2019), upregulation of GPX4 (Park et al., 2019), and overexpression of SLC7A11 to increase GSH levels (Fang et al., 2020) are capable of inhibiting ferroptosis in cardiomyocytes.

Iron chelators are able to block redox reactions catalyzed by iron ions and to allow efficient transport and excretion without iron redistribution. Nowadays, 3 types of iron chelators including deferiprone, deferoxamine, and deferasirox are applied in clinical practice, mostly for the treatment of IOC (Pennell et al., 2013). Compared to the other two iron chelators, deferiprone targets hemorrhage-derived iron in IRI, which exerts a cardioprotective effect in acute MI by alleviating intramyocardial hemorrhage and cardiac hypertrophy (Behrouzi et al., 2020). Moreover, several retrospective studies revealed that deferiprone monotherapy showed better protection in heart than deferoxamine therapy or subcutaneous desferrioxamine therapy (Galanello et al., 2006; Pepe et al., 2011; Bilgin et al., 2020; Li et al., 2021).

The cardioprotective effects of antioxidants (e.g., Fer-1, liproxstatin-1, vitamin E) have been verified currently. Fer-1 is demonstrated to eliminate alkoxyl radicals produced by Fe^2+^ from lipid hydroperoxides in other excess iron-induced ferroptosis (Baba et al., 2018). Also, Fer-1 is beneficial to cardiomyopathy in Fth-deficient mice (Fang et al., 2020). Likewise, liproxstatin-1 can decrease the levels of voltage-dependent anion channel 1 and rescue GPX4 levels to protect myocardium against IRI (Feng et al., 2019). Vitamin E and α-tocotrienol are capable of inhibiting ferroptosis by suppressing LOXs (Kagan et al., 2017).

## Perspective and Conclusion

In this review, we summarized the main mechanisms of ferroptosis and discussed the role of ferroptosis in CVDs. As CVDs are a global health problem causing high rates of mortality, morbidity, and disability, understanding the pathology of cardiomyocyte damage is essential to develop a promising and novel therapeutic strategy for CVDs. With more and more focus on RCD in cardiomyocytes, ferroptosis, as an iron-dependent form of cell death, has received increasing attention.

Ferroptosis is mainly caused by the occurrence of lipid peroxidation of PUFA accumulation, which results from the accumulation of intracellular free Fe^2+^ and/or dysfunction of GSH peroxidation. Hence, the prominent features of ferroptosis are PUFA or PL peroxidation as well as accumulation of excessive iron. With overload of iron and lipid peroxidation, ROS accumulates and cell membrane is damaged, which eventually leads to cell death.

Iron overload is recently proved to be the significant cause of cardiomyocyte death, with cardiovascular imaging showing accumulation of iron in the damaged zone of heart. Also, verified by abundant studies with specific models *in vivo* and *in vitro*, ferroptosis has been demonstrated to play an important role in different types of CVDs, including cardiomyopathy, MI, IRI, HF, vascular injury, stroke, arrhythmia and so on. Inhibition of ferroptosis in CVDs can decrease cardiomyocyte death and improve cardiopathic conditions. Consequently, ferroptosis is a promising therapeutic target for CVDs.

However, the molecular mechanisms of ferroptotic cell death in cardiomyocytes remain unclear. Except for the destruction of iron metabolism, GSH depletion and lipid peroxidation, various pathways are also involved in the process of ferroptosis, such as high levels of extracellular glutamic acid, organelle-mediated pathways, Nrf2 pathway and so on (Zhai et al., 2021). Moreover, there are few researches on the relationship between ferroptosis and pathogenesis of arrhythmia. And further studies need to be performed to demonstrate the association between ferroptosis and vascular diseases. Though several inhibitors of ferroptosis, such as Fer-1 and GPX4, have been proposed to effectively repair cardiomyocyte injury, these novel methods are unsuitable for routine clinical therapy and their feasibility needs verification.

In conclusion, ferroptosis plays a significant role in the pathogenesis of various CVDs. With mechanisms and clinical feasibility under exploration, targeting ferroptosis to treat CVDs is a new continent to be explored.

## Author Contributions

FH, PYZ, JL, and SZ contributed to the conception and design of the study. FH, RY, and ZX searched the literature and wrote the manuscript. YX, XL, and PZ created the figure and table, and participated in drafting the manuscript. PYZ, JL, and SZ revised the manuscript. All the authors read and approved the final manuscript.

## Conflict of Interest

The authors declare that the research was conducted in the absence of any commercial or financial relationships that could be construed as a potential conflict of interest.

## Publisher’s Note

All claims expressed in this article are solely those of the authors and do not necessarily represent those of their affiliated organizations, or those of the publisher, the editors and the reviewers. Any product that may be evaluated in this article, or claim that may be made by its manufacturer, is not guaranteed or endorsed by the publisher.
